# Supplementary material for: "Fear of the unknown": Health, disability, and stakeholder perspectives on the behavioral and social drivers of vaccination in children with disability in Fiji
Source: PLOS Glob Public Health. 2025 Jan 8;5(1):e0004132. doi: 10.1371/journal.pgph.0004132 (PMC11709282; doi:10.1371/journal.pgph.0004132)
Supplement: S1 Appendix — (DOCX) [file pgph.0004132.s002.docx]

**S1 Appendix**

**"Fear of the unknown": Health, disability, and stakeholder perspectives on the behavioral and social drivers of vaccination in children with disability in Fiji.**

**Focus Group Interview Guides**

**Health workers and stakeholders**

1. Could you each please introduce yourselves and tell us about the work you do in immunization with children with disability?

*Probes:*

- *What are you responsible for?*
- *How many days do you work in this role?*
- *Where do you perform your duties?*

1. To what extent does your role involve immunization?

*Probes:*

- *What parts of your job involve immunization?*
- *Can you tell me more about that?*

1. I’d like to understand the process involved in immunizing a child with disability/ special needs. Can you tell me about this starting from the very beginning?” OR “What is involved in immunizing a child with disability/ special needs?”

*Probes:*

- *Does it involve work for you even before the family arrives at the centre for vaccination?*
- *Can you summarize the immunization procedure in around five steps, starting once a family arrives at the center for vaccination? [Note: Adjust this question for non-clinic settings if required.]*
- *Are there follow-ups or steps involved once they leave the center? [Note: Other probes, such as ongoing door-to-door, systems of recording vaccinations, making vaccination cards, and so on, could be added as required.]*

1. What do you find difficult when it comes to helping families stay up to date with immunization?

*Probes:*

- *Which part of the process you described before do you find the hardest to complete? Why is that?*
- *Can you give some examples of reasons people give when their child with disability has fallen behind the vaccination schedule?*
- *Can you give some examples of reasons that people give for refusing vaccines for their children with disability?*

1. What do you find works in helping families stay up to date with immunization?

*Probes:*

- *What helps them not to miss doses or appointments? [Note: This is to probe for practical issues.]*
- *What helps those who are hesitant about getting their children vaccinated?*

1. If you had the chance, what would you do to improve immunization services in your area?

**Disability and community stakeholders**

1. Could you each please introduce yourselves including telling me about the work you do in relation to immunization/ injection with children with disability/ special needs?

*Probes:*

- *What are you responsible for?*
- *How many days do you work in this role?*
- *Where do you perform your duties?*

1. To what extent does your role involve immunization/ injection?

*Probes:*

- *What parts of your job involve immunization/ injection?*
- *Can you tell me more about that?*

1. I’d like to understand the process involved in immunizing a child with disability/ special needs. Can you tell me about this starting from the very beginning?” OR “What is involved in immunizing a child with disability/ special needs?”

*Probes:*

- *Do you visit each household and inform parents about the health team visitation?*
- *Do you keep track of the children’s immunization record in your community?*
- *Do you assist parents/ guardians of children with disability by taking them to the health center/ hospital when the child is due for vaccination?*

1. How much do you contribute to and participate in national vaccination programs and goals? How well do you think routine immunization services are delivered?

*Probes:*

- *Do you provide access to underprivileged or remote groups that would not otherwise have access to immunizations?*
- *Are health worker behaviors different, such as spending more time with clients explaining vaccination? Are health workers more motivated, and does this impact routine vaccination?*
- *Does your current position generally affect the level of trust in the areas in which you operate (and how does this relate to immunization services or uptake)?*
- *Do the communities where you operate have different access to public services?*
- *Are primary healthcare services and outreach efforts any more or less effective?*

1. What do you find difficult when it comes to helping families stay up to date with immunization?

*Probes:*

- *Which part of the process you described before do you find the hardest to complete? Why is that?*
- *Can you give some examples of reasons people give when their child has fallen behind the vaccination schedule?*
- *Can you give some examples of reasons that people give for refusing vaccines for their children?*

1. What do you find works in helping families stay up to date with immunization?

*Probes:*

- *What helps them not to miss doses or appointments? [Note: This is to probe for practical issues.]*
- *What helps those who are hesitant about getting their children vaccinated?*

7. If you had the chance, what would you do to improve immunization services in your area?
