# Supplementary material for: "Fear of the unknown": Health, disability, and stakeholder perspectives on the behavioral and social drivers of vaccination in children with disability in Fiji
Source: PLOS Glob Public Health. 2025 Jan 8;5(1):e0004132. doi: 10.1371/journal.pgph.0004132 (PMC11709282; doi:10.1371/journal.pgph.0004132)
Supplement: Attachment — Submitted filename: Review Comments] [Sep 30.doc [file pgph.0004132.s003.doc]

**REVIEWER REPORT**

**TOPIC**

"Fear of the unknown": Health, disability, and stakeholder perspectives on the behavioral and social drivers of vaccination in children with disability in Fiji.

**Remark(s) 1:** Remove the full stop from the topic, otherwise, it reads fine.

**ABSTRACT**

**Background:**

**Page 3, sentence 2, lines 33-35:** Understanding the attitudes and behaviors of health, disability, and community stakeholders toward vaccination uptake in these children is important, as they can impact the success of vaccination programs.

**Remark(s) 2:** Recast to read: “…uptake among children is important, as they can impact the success of vaccination programs.”

**Remark(s) 3:** Remove all abbreviations from the abstract, there is no need for them.

**Conclusions:**

**Page 3, lines 50-53:** The findings from this study can guide strategic action….

**Remark(s) 4:** This is not a good conclusion, recast.

**INTRODUCTION**

**Page 5, para2, lines 84-87:** To better understand access to immunization among children with disability in Fiji and to inform the present study, we conducted a cross-sectional survey to examine vaccination rates in children with disability. We found that only 55% of children with disability in our study location were vaccinated against basic antigens.16

**Remark(s) 5:** This portion is problematic. Did the authors conduct this previous study in furtherance of the current study? This portion should be reported under the methods given the link with the current study. I surmise the authors are trying to build a strong justification for the current study, however, the presentation is not clear. I suggest a complete rephrase of this portion of the problem statement.

**MATERIALS AND METHODS**

**Study location:**

**Page 5, lines 101 & 102:** This study was conducted in peri-urban and urban areas of the Suva–Nausori corridor in Fiji.

**Remark(s) 6:** This is inadequate in providing details on the location.

**Design and theoretical framework**

**Page 6, lines 108-113:** We used the World Health Organization’s (WHO) Behavioral and Social Drivers (BeSD) of Vaccination Framework20. A 12-member stakeholder group was actively engaged and involved throughout all stages of the project providing expertise to guide the study design, data collection, analysis, and dissemination of results. The group included local and international disability and health service providers (study partners), MHMS, academics, and community and faith leaders.

**Remark(s) 7:** There are no details on the design and theoretical framework provided; reorganize this sub-section. This can undermine the reproducibility of the study. Moreover, what is the justification for the choice of design? You need to justify this with appropriate sources.

**Recruitment and participants:**

**Page 6, lines 115-128:** Immunization health workers, disability service providers, and advocates from local non-government….

**Remark(s) 8:** There are issues with this sub-section. First, there was no source to support the sampling technique. Second, what is the size of the target population? What are the exclusion criteria?

**Procedure:**

**Page 7, lines 131-134:** Two focus group interview guides (one for immunization healthcare workers and another for disability and community stakeholders) were adapted from the WHO BeSD childhood vaccination qualitative framework analysis template tool….

**Remark(s) 9:** If the interview guide was adapted, then you need to provide details on what was added or removed or varied in the WHO BeSD tool. Meanwhile, you reported earlier under the design and theoretical framework that you used the World Health Organization’s (WHO) Behavioral and Social Drivers (BeSD) of Vaccination Framework. You must be consistent on how the instrument was developed.

**Data analysis**

**Page 8, lines 154-174:** A combination of inductive and deductive reflexive thematic analysis was ….

**Remark(s) 10:** How did you ensure qualitative rigour?

**RESULTS**

**Remark(s) 11:** While the section provided a detailed account of the themes that emerged from the data analysis, the authors failed to show whether the findings were expected or not. At least, the authors must go beyond the mere presentation of the findings and provide their own assessment of same by clearly demonstrating whether the findings were surprising or not.

**LIMITATIONS**

**Remark(s) 12:** Provide a section for limitations of the study

**COMMENTS FOR AUTHORS**

Generally, the authors did well in exploring an area of public health that is yet to receive the needed research attention. Overall, the paper was well written and the authors have demonstrated a good knowledge of the subject matter. However, there are observations needing proper resolution by the authors to improve the quality of the paper and make it more beneficial for the global readership. Specific comments on observations made have been provided above.

**COMMENTS FOR THE EDITOR**

Though the authors have made a good effort at producing the study, there are issues that must be resolved to guarantee the overall quality of the paper. At the current state, the paper would need substantial revision to make it suitable for publication.
