# Supplementary material for: "Fear of the unknown": Health, disability, and stakeholder perspectives on the behavioral and social drivers of vaccination in children with disability in Fiji
Source: PLOS Glob Public Health. 2025 Jan 8;5(1):e0004132. doi: 10.1371/journal.pgph.0004132 (PMC11709282; doi:10.1371/journal.pgph.0004132)
Supplement: Attachment — Submitted filename: PLOS Global Public Health - reviewer report Fear of Unknown.docx [file pgph.0004132.s004.docx]

**PLOS Global Public Health "Fear of the unknown": Health, disability, and stakeholder perspectives on the behavioral and social drivers of vaccination in children with disability in Fiji - PGPH-D-24-01875**

***Reviewer’s Report***

The study is relevant and will add to the body of knowledge. The authors have rightly pointed out that vaccine coverage among the healthy population is a challenge, so therefore, vaccination uptake among children with disability/disabilities, particularly living in middle- and low-income countries face manifold challenges.

The following are my comments on this paper:

1. Line 33 – 34 is not clear – “Understanding the attitudes and behaviors of health, disability, and community stakeholders towards vaccination uptake in these children is important. – Whose attitudes and behaviors? Compare Lines 96 – 98 and 104 and reword this sentence to reflect clearly that the perspectives of the stakeholders are being examined as stated in your topic of the study.
2. Line 83 – what was the reason for the recovery vaccination coverage in 2023? What did the literature state, need expansion on this statement.
3. Line 105 – there should be a period after the word ‘disability’ marking the end of the sentence. Otherwise, it is confusing to the reader.
4. Line 106 – mention where and under which section the research design for the first part is explained. It is very vague.
5. Line 108 – Under Design and Theoretical Framework, no comprehensive research design of the present study was stated or any theoretical framework included. This section needs more elaboration on the research design and theoretical framework. Only Vaccination Framework was mentioned but that too need some information for the readers who are not familiar with this framework.
6. Line 199 – What is meant by the phrase “plenty coconut wireless around”? We know that with respect to the dissemination of information and misinformation during COVID-19 was rampant through all platforms of communication that cause fears of the “unknown’ among the population. So, what was the scenario prior to COVID-19 in terms of information relating to vaccination, what misunderstanding or miscommunication if any prevailed during that time? Did you find any literature that can be included here or in the introduction or discussion section.
7. Line 218 – with reference to this statement, did you investigate what perspectives stakeholders held prior to COVID-19, did COVID experience escalate the fear of the unknown with regards to vaccination among children with disabilities?
8. Line 435 – 436 – is there any literature to support this finding?
9. Discussion Section – should address all major themes of the study and linkages between these themes should be evident – thinking and feeling, social process, motivation, practical – need to revisit what you stated in the introduction with regards to these themes.
