# Supplementary material for: "Fear of the unknown": Health, disability, and stakeholder perspectives on the behavioral and social drivers of vaccination in children with disability in Fiji
Source: PLOS Glob Public Health. 2025 Jan 8;5(1):e0004132. doi: 10.1371/journal.pgph.0004132 (PMC11709282; doi:10.1371/journal.pgph.0004132)
Supplement: Attachment — Submitted filename: Reply to Reviewers_241125.docx [file pgph.0004132.s005.docx]

Thank you for the opportunity to revise and re-submit. We have responded to each of the reviewer comments point-by-point below and updated the manuscript accordingly. A version of the manuscript with tracked changes, and a clean version, have been uploaded. We outline our responses to the reviewers below.

# Reviewer #1

TOPIC

1. "Fear of the unknown": Health, disability, and stakeholder perspectives on the behavioral and social drivers of vaccination in children with disability in Fiji.
   **Remark(s) 1:** Remove the full stop from the topic, otherwise, it reads fine.
   **Response:** Thank you, the full stop has now been removed.

ABSTRACT

2. Background: Page 3, sentence 2, lines 33-35: Understanding the attitudes and behaviors of health, disability, and community stakeholders toward vaccination uptake in these children is important, as they can impact the success of vaccination programs.

**Remark(s) 2:** Recast to read: “…uptake among children is important, as they can impact the success of vaccination programs.”

**Response:** Changes to this sentence were also requested by reviewer #7. It has now been edited to, “This study aimed to understand the social and behavioral drivers of vaccine uptake among children with disability in Fiji, from the perspectives of health, disability and community stakeholders”.

3. **Remark(s) 3:** Remove all abbreviations from the abstract, there is no need for them.
   **Response:** Abbreviations have now been removed from the abstract.
4. Conclusions: Page 3, lines 50-53: The findings from this study can guide strategic action….
   **Remark(s) 4:** This is not a good conclusion, recast.

**Response:** The conclusion has now been revised to:

(page 3) *The findings from this study can inform strategic actions to overcome barriers to vaccination for children with disability, including strengthening existing vaccination programs, promoting greater equity in vaccination for children with disability in Fiji. This will reduce the burden of vaccine-preventable diseases in this priority group.*

INTRODUCTION

5. Page 5, para2, lines 84-87: To better understand access to immunization among children with disability in Fiji and to inform the present study, we conducted a cross-sectional survey to examine vaccination rates in children with disability. We found that only 55% of children with disability in our study location were vaccinated against basic antigens.16
   **Remark(s) 5:** This portion is problematic. Did the authors conduct this previous study in furtherance of the current study? This portion should be reported under the methods given the link with the current study. I surmise the authors are trying to build a strong justification for the current study, however, the presentation is not clear. I suggest a complete rephrase of this portion of the problem statement.
   **Response:** Yes, the cross-sectional survey to examine vaccination rates in children with disability was conducted in furtherance of the current study. The results from the cross-sectional survey are published elsewhere. We have edited the introduction to help clarify the relationship between the two parts of the broader research project.

(page 6) *To better understand access to immunization among children with disability in Fiji and to inform the present study, we conducted a cross-sectional survey to examine vaccination rates in children with disability. We found that only 55% of children with disability in the study location (the same location as the present study) were vaccinated against basic antigens.*

We have also edited the ‘Materials and Methods’ section to clarify that the current paper is part of a broader research project involving two parts (1) cross-sectional survey estimating vaccine uptake among children with disability; and (2) focus group interviews with health, disability, and community stakeholders on the behavioral and social drivers of vaccination uptake among children with disability. In this paper, we report only on the qualitative data from the focus groups. We have moved this information to ‘Design and theoretical framework’:

(page 7) *The study is a two-part study looking at immunization in children with disability. Part 1 involved a cross-sectional survey examining vaccine uptake among 198 children with disability and their caregivers (published elsewhere, see ^1^). Part 2 involved a qualitative study utilizing focus groups with health, disability, and community stakeholders on the behavioral and social drivers of vaccination uptake among children with disability. In this paper, we present qualitative data from the focus groups.*

MATERIALS AND METHODS

6. Study location: Page 5, lines 101 & 102: This study was conducted in peri-urban and urban areas of the Suva–Nausori corridor in Fiji.

**Remark(s) 6:** This is inadequate in providing details on the location.
**Response:** Thank you, we have now re-worked this section to provide additional information about the study location:

(page 8) *This study was conducted in peri-urban and urban areas of the Suva–Nausori corridor. A 19km stretch between Suva (capital of Fiji) and Nausori on its north east. The study site was chosen in collaboration with Fiji's Ministry of Health and Medical Services and non-governmental partners to ensure representation from both rural and urban areas. Based on in-country unpublished data it is believed to be an area with high prevalence of functional impairments among children (6% in ages 2-4 and 9% in ages 5-17)^2^, as well high population density (approximately 164,000)^3^, and challenging access to routine healthcare services.*

*Design and theoretical framework*

7. Page 6, lines 108-113: We used the World Health Organization’s (WHO) Behavioral and Social Drivers (BeSD) of Vaccination Framework20. A 12-member stakeholder group was actively engaged and involved throughout all stages of the project providing expertise to guide the study design, data collection, analysis, and dissemination of results. The group included local and international disability and health service providers (study partners), MHMS, academics, and community and faith leaders.
   **Remark(s) 7:** There are no details on the design and theoretical framework provided; reorganize this sub-section. This can undermine the reproducibility of the study. Moreover, what is the justification for the choice of design? You need to justify this with appropriate sources.

**Response:** Thank you for noting this; we welcome the opportunity to explain our study design and theoretical framework in more detail. We have now reorganized and expanded this section, including moving some content that was previously under ‘study location’ and providing additional references:

(page 7) *The study is a two-part study looking at immunization in children with disability. Part 1 involved a cross-sectional survey examining vaccine uptake among 198 children with disability and their caregivers (published elsewhere, see ^1^). Part 2 involved a qualitative study utilizing focus groups with health, disability, and community stakeholders on the behavioral and social drivers of vaccination uptake among children with disability. In this paper, we present qualitative data from the focus groups.*

*A 12-member stakeholder group was actively engaged and involved throughout all stages of the project providing expertise to guide the study design, data collection, analysis, and dissemination of results.^4, 5^ The group included local and international disability and health service providers (study partners), MHMS, academics, and community and faith leaders. The stakeholder group identified focus groups as the preferred method for data collection, as participants were anticipated to feel more comfortable sharing their opinions in a group setting, reducing the pressure of individual interviews or a survey, and enabling them to control their level of participation.^6^ Additionally, the group setting fosters interaction among participants, encouraging the exchange of ideas and yielding a deeper, richer understanding of sensitive and potentially taboo topics.^6^*

*We used the World Health Organization’s (WHO) Behavioral and Social Drivers (BeSD) of Vaccination Framework^7^ to provide a structured approach to understanding the complex factors influencing vaccination uptake among children with disability. This framework offered a comprehensive guide to examine not only individual beliefs and behaviors but also social, cultural, and systemic drivers that can impact vaccine acceptance, access and uptake. This framework is well-suited for informing strategies to improve equitable vaccine uptake in contexts with diverse social and healthcare challenges.^7^*

Providing details on how the framework was developed are beyond the scope of this paper and can be found in its original source. Additional details on the WHO BeSD Framework are available here: <https://www.who.int/publications/i/item/who-wer9720-209-224>

*Recruitment and participants*

8. Page 6, lines 115-128: Immunization health workers, disability service providers, and advocates from local non-government….
   **Remark(s) 8:** There are issues with this sub-section. First, there was no source to support the sampling technique. Second, what is the size of the target population? What are the exclusion criteria?

**Response:** Thank you for your feedback. We have now included a reference to support our sampling method.

*Campbell S, Greenwood M, Prior S, Shearer T, Walkem K, Young S, et al. Purposive sampling: complex or simple? Research case examples. J Res Nurs. 2020;25(8):652-61.*

In reflexive thematic analysis, the concept of a target population size is not directly applicable, as the goal is not statistical representation but rather to achieve information power by gathering in-depth insights from a diverse range of perspectives. Our sampling aimed to include a variety of health, disability, and community stakeholders relevant to the study's focus on vaccination uptake among children with disabilities. For this reason, an exclusion criterion is not appropriate for the focus groups.

*Procedure*

Page 7, lines 131-134: Two focus group interview guides (one for immunization healthcare workers and another for disability and community stakeholders) were adapted from the WHO BeSD childhood vaccination qualitative framework analysis template tool….
**Remark(s) 9:** If the interview guide was adapted, then you need to provide details on what was added or removed or varied in the WHO BeSD tool. Meanwhile, you reported earlier under the design and theoretical framework that you used the World Health Organization’s (WHO) Behavioral and Social Drivers (BeSD) of Vaccination Framework. You must be consistent on how the instrument was developed.
**Response:** The WHO BeSD childhood vaccination is a validated tool for use in countries to measure social and behavioral drivers for vaccination (see here: https://www.who.int/publications/i/item/who-wer9720-209-224) . The tool was adapted based on in-country investigators’ feedback and pilot testing. For example, additional prompts were added asking about immunization for underprivileged or remote populations, as well as outreach initiatives such as door-to-door services. Specific modifications were made to address the vaccination needs of children with disability/ special needs. The adapted tools are provided in the Supplementary material, Appendix 1. The manuscript has now been edited to state:

(page 9) *Two focus group interview guides (one for immunization healthcare workers and another for disability and community stakeholders) were adapted from the WHO BeSD childhood vaccination qualitative framework analysis template tool (Annex 1, WHO BeSD Vaccination Framework^7^). The adaptation process was conducted in collaboration with the in-country investigator team and stakeholder group, followed by pilot testing with two groups to confirm feasibility. Modifications included incorporating local terminology, improving in-depth probing, and asking specifically about the vaccination of ‘children with disability or special needs’. Additional prompts were introduced to evaluate immunization efforts targeting underprivileged or remote populations and outreach strategies such as door to door services, see S1 Appendix.*

*Data analysis*

9. Page 8, lines 154-174: A combination of inductive and deductive reflexive thematic analysis was ….
   **Remark(s) 10:** How did you ensure qualitative rigor?

**Response:** Thank you for your question regarding how we ensured qualitative rigor in our analysis. As detailed in the manuscript, we adopted a comprehensive approach to maintaining rigor throughout the reflexive thematic analysis process, some examples being:

- Rationale for data generation methods: We have expanded on our reasoning for using focus groups as the primary data generation method.
- Characteristics of data generation tools: The focus group schedules are provided in the supplementary material, and we have described the piloting process for these tools.
- Researcher engagement with the analysis process: Our analysis involved regular discussions among research team members to critically challenge our interpretations and foster a collaborative approach to theme generation. This process ensured that multiple perspectives were considered, enhancing the robustness of our findings. Additionally, we committed to an extended engagement with the data, immersing ourselves in the transcripts to fully understand the nuances of participants' experiences. This thorough familiarity with the data allowed us to identify subtle themes and patterns.
- Preparation of data for analysis: All interviews were transcribed verbatim, and the accuracy of these transcriptions was verified by two independent researchers. This step minimized errors and ensured that participants’ voices were accurately captured, which is crucial for credibility.
- Evidence of reflexive practice: Throughout the analysis, we actively engaged in reflexivity, maintaining awareness of our roles in knowledge production. We critically reflected on how our cultural backgrounds, ages, genders, and other intersectional characteristics shaped our assumptions and influenced the research process. This reflective practice helped us to mitigate biases and acknowledge our positionality in relation to the research. We discuss this in the methods and discussion sections.

We have also carefully reviewed our reporting against the recently published, Reflexive Thematic Analysis Reporting Guidelines (RTARG) (<https://doi.org/10.1177/02692163241234800>). We are confident that our research was conducted and reported in line with best practices in reflexive thematic analysis, ensuring both rigor and transparency throughout the study. Our analysis was systematic and thorough, allowing us to generate well-substantiated findings that accurately reflect participants' perspectives.

RESULTS

10. **Remark(s) 11:** While the section provided a detailed account of the themes that emerged from the data analysis, the authors failed to show whether the findings were expected or not. At least, the authors must go beyond the mere presentation of the findings and provide their own assessment of same by clearly demonstrating whether the findings were surprising or not.

**Response:** Thank you for your feedback regarding our reporting of the results. We appreciate your suggestion for a more in-depth analysis of whether the findings were expected or surprising. However, we would like to clarify that the primary purpose of this paper's results section is to present our study's findings without interpretation or subjective assessment. In our manuscript, we have adhered to this structure by clearly delineating the results from the discussion. The discussion section is intended to provide a comprehensive analysis of the findings, including our interpretations and comparisons to previous literature. For example, we mention – “The relationship between vaccine uptake and safety concerns is well established in general vaccine literature” So we are unsure as to what the reviewer means by results by their comment. If there is a specific question the reviewer – we are happy to answer that. We believe that the approach we have used allows for a clear and organized presentation of information, ensuring that readers can easily distinguish between the reported findings and the subsequent analysis and implications.

LIMITATIONS

11. Remark**(s) 12:** Provide a section for limitations of the study.

**Response:** Thank you, we have revised the discussion section to address limitations more clearly:

(page 25) *This study has several limitations. First, our focus was specifically on immunization and disability stakeholders which meant we did not collect qualitative data from children with disability and their families. While this approach allowed us to gather insights directly from those influencing vaccination policies and practices, it limits our understanding of the experiences and perspectives of the children and families most affected by these issues. The focus groups were also small, which may restrict the diversity of viewpoints captured. Furthermore, while our research examined vaccine uptake and social and behavioral drivers using the WHO BeSD survey tools, future research should examine effective interventions to increase vaccine uptake in this population. These approaches should be co-designed with children with disability and their families to address barriers to vaccination specific to children with disability in LMICs.^8^*

12. COMMENTS FOR AUTHORS

Generally, the authors did well in exploring an area of public health that is yet to receive the needed research attention. Overall, the paper was well written and the authors have demonstrated a good knowledge of the subject matter. However, there are observations needing proper resolution by the authors to improve the quality of the paper and make it more beneficial for the global readership. Specific comments on observations made have been provided above.

**Response:** Thank you for your thoughtful and detailed review of our paper. We have aimed to address each of your comments above.

# Reviewer #2

13. A well written manuscript. The results adequately support the authors' claims regarding the challenges faced by disabled children in Fiji regarding their right to access to care.
    The methodology, which combines a cross-sectional survey with focus groups provides a valuable mixed methods design of both vaccination rates and stakeholder's perspectives. The sampling strategy is appropriate. The use of the WHO BeSD Framework provides a strong theoretical underpinning for the study and the iterative process of thematic analysis by a number of researchers, enhances the reliability and validity of the findings. More attention could be given to the information provided about the potential biases and their mitigation in both data collection and analysis. The recommendations offer practical implications for health policy improvements for this population and provide ideas for actionable interventions. In summary, this manuscript presents a well-structured study that contributes valuable knowledge to understanding vaccination disparities among children with disabilities in Fiji. The results are compelling, methodologies sound, ethical considerations robust, and conclusions appropriately derived from the findings.
    **Response:** Thank you for your thoughtful feedback, particularly regarding the need for greater attention to potential biases and their mitigation in both data collection and analysis. We recognize the importance of transparency in addressing these issues to enhance the rigor of our research. In our response to Reviewer 1 (remark 10), we provided a detailed explanation of how we ensured rigor in accordance with the Reflexive Thematic Analysis Reporting Guidelines (RTARG). Additionally, we worked closely with a 12-member stakeholder group that included individuals with diverse backgrounds, experiences, and perspectives, which helped mitigate individual biases and enrich our analysis.

# Reviewer #3

14. The manuscript entitled "Fear of the unknown": Health, disability, and stakeholder perspectives on the behavioral and social drivers of vaccination in children with disability in Fiji" is an essential study for vulnerable groups, and the research topic is critical as all children with disabilities should be vaccinated and prevented from vaccine-preventable diseases. The study presents important findings through a qualitative study using five focus group discussions on what hinders children with disabilities from getting vaccinated. They found that stigma, low knowledge of the benefits of vaccines, misinformation, and inaccessible health service delivery are the factors that hinder children with a disability from getting vaccinated. […] Overall, the paper is fantastic and easy to read. Thank you.

**Response:** Thank you

15. The paper is well written except for a few typo errors, such as on Page 5, Line 88, the word "knows" should be "known," and on Page 6, Line 105, there should be a period to separate two sentences.

**Response:** Thank you, these typos have now been corrected.

Page 5, line 88: “knows” has now been corrected to “known”

Page 6, line 105: a period has now been entered to separate the two sentences.

16. I am only concerned about the use of FGD as a data collection technique, where the number of participants in some FGDs was less than four. Hence, justifying the need for FGD for this study would improve the paper.

**Response:** Thank you for this comment. We have now provided additional information in the Methods section to explain the use of focus groups in this study.

(page 7) *The stakeholder group identified focus groups as the preferred method for data collection, as participants were anticipated to feel more comfortable sharing their opinions in a group setting, reducing the pressure of individual interviews or a survey, and enabling them to control their level of participation.^6^ Additionally, the group setting fosters interaction among participants, encouraging the exchange of ideas and yielding a deeper, richer understanding of sensitive and potentially taboo topics.^6^*

We have now also acknowledged this concern in the new limitations section in the Discussion.

(page 25) *The focus groups were also small, which may restrict the diversity of viewpoints captured.* *Fiji also has a small workforce^9^ which meant that there are only a small number of experts and stakeholders who can be consulted.)*

17. Similarly, I could not see much text on the trustworthiness of the findings. I think explaining about it could benefit the paper.

**Response:** Thank you for identifying this important point. In our response to Reviewer 1 (remark 10), we offered a comprehensive explanation of how we ensured the rigor and trustworthiness of our findings, in alignment with the Reflexive Thematic Analysis Reporting Guidelines (RTARG). Furthermore, we collaborated closely with a 12-member stakeholder group composed of individuals with diverse backgrounds, experiences, and perspectives. This collaboration not only helped mitigate individual biases but also enriched our analysis.

# Reviewer #4

The manuscript offers a valuable contribution to the understanding of the behavioural and social factors influencing vaccine uptake among children with disabilities in Fiji, as perceived by key stakeholders. It is emphasised that there is a necessity to diminish the prevalence of preventable illnesses among disabled children in Fiji. Nevertheless, a number of points require further clarification and refinement.

18. Abstract:
    While the study offers valuable insights, However, the abstract is not sufficiently precise. It is recommended that the authors refine the writing of these two subsections, namely 'Context' and 'Methods'.
    For instance, the qualitative research approach employed in this study should be explicitly delineated in the 'methodology' subsection, while the study's objective should be clearly articulated in the 'context' subsection.
    **Response:** Thank you, we have edited the Abstract to clearly identify the study’s objective in the ‘background’ section and we have mentioned ‘qualitative’ and ‘reflexive thematic analysis’ in the methods section:

(page 3) *Background:* *Vaccinating children with disability in low- and middle-income countries, such as Fiji, is a key priority for equity. This study aimed to understand the social and behavioral drivers of vaccine uptake among children with disability in Fiji, from the perspectives of health, disability, and community stakeholders.*

*Method: Five qualitative focus groups were conducted with 22 stakeholders, including healthcare workers, disability service providers and advocates, and community and faith leaders (female n=17, 77%). Data were collected and analyzed using reflexive thematic analysis and applied the World Health Organization’s Behavioral and Social Drivers of Vaccination framework.*

19. Methods:
    It is recommended that the authors limit the scope of the study to the essential elements.
    Furthermore, the authors should provide clarification regarding the qualitative research approach employed. The text could be deleted or made more fluid by replacing it with the following: 'The study consisted of two parts:
    'A) A survey examining vaccination among 198 disabled children and their carers; B) focus groups with health, disability and community stakeholders on behavioral and social factors of vaccination among disabled children.
    This is because the authors state in the 'Introduction' section that they conducted an initial quantitative study (cf. last paragraph of the 'Introduction' section, from 96-101).
    It is therefore difficult to understand the design of the current study.

**Response:** Thank you, we have now edited this section to clarify the broader study:

(page 7) *The study is a two-part study looking at immunization in children with disability. Part 1 involved a cross-sectional survey examining vaccine uptake among 198 children with disability and their caregivers (published elsewhere, see ^1^). Part 2 involved a qualitative study utilizing focus groups with health, disability, and community stakeholders on the behavioral and social drivers of vaccination uptake among children with disability. In this paper, we present qualitative data from the focus groups.*

# Reviewer #5

20. This study sheds light on the often-overlooked barriers to vaccination for children with disabilities in Fiji. The title reflects the key themes, but it is too long.

**Response:** Thank you for identifying this concern. However, we have decided to retain the title as it accurately describes the study.

21. The introduction highlights the importance of vaccination in reducing childhood morbidity and mortality but could be more concise, especially in discussing vaccination campaigns.
    **Response:** It is unclear what exactly the reviewer is seeking in this section. Since none of the other reviewers have requested this, we have not made major edits. We think it is important to retain the information about Fiji’s immunization program to provide context and background as we cannot assume that the readers will have prior knowledge of immunization globally and in Fiji.
22. The term "intersectional inequality" is not fully unpacked for readers unfamiliar with its complexities, especially in the specific context of disabilities and vaccination.
    **Response:** We have edited this sentence to better engage with readers unfamiliar with the term ‘intersectional inequality’:

(page 5) *This is because children with disability often face multiple barriers to accessing vaccination due to the impacts of disability, poverty, and inequity in access to basic primary healthcare services.*

23. The recruitment process is clear, but clarify the roles between different groups (e.g., health workers, disability providers, and advocates) by specifying their unique roles in the study.
    **Response:** This study brought together diverse groups - healthcare workers, disability service providers and advocates, and community and faith leaders - because of their distinct roles in the lives of children with disability and unique perspectives on behavioral and social drivers to vaccination uptake for this population.

The roles of these participant groups are also discussed in the introduction (page 6):

*It is well known that healthcare providers play a crucial role in addressing these barriers and influencing both decision-making and access to immunization for children with disability.^10, 11^ Additionally, in Fiji, stakeholders outside of the healthcare system, including disability and community stakeholders (e.g., education providers and those in faith leadership roles), are instrumental in shaping public perceptions and attitudes to vaccination and influencing health behaviors for people with disability.^9, 12, 13^ Therefore, to increase vaccination coverage, it is essential to understand the attitudes and examine the perspectives of multiple stakeholder about the vaccination of children with disability and the factors that enable and prevent vaccine uptake in this population.^7^*

24. The mention of managing power dynamics is good, but explain how grouping by hierarchical position achieves this.

**Response:** Thank you, we have now expanded our explanation for this decision:

(page 9) *On the advice of our stakeholder group, participants working at the same organization were allocated to separate focus groups, while people of similar hierarchical positions were grouped together to manage power dynamics. This approach allowed participants to engage more candidly without concerns about authority or judgement from higher-ranking colleagues and minimized the risk of self-censorship and inhibited discussion.*

25. The procedure section is detailed. The process of adapting the interview guides from the WHO BeSD framework could be better explained to show how local relevance was incorporated.
    **Response:** Thank you for this comment. We have now provided additional detail about the adaptation process, as outlined our response to reviewer 1, remark #9. The manuscript has now been edited to state:

(page 9) *Two focus group interview guides (one for immunization healthcare workers and another for disability and community stakeholders) were adapted from the WHO BeSD childhood vaccination qualitative framework analysis template tool (Annex 1, WHO BeSD Vaccination Framework^7^). The adaptation process was conducted in collaboration with the in-country investigator team and stakeholder group, followed by pilot testing with two groups to confirm feasibility.*

26. In-depth probing was improved after the pilot test, but more detail on the specific changes made would add clarity.
    **Response:** Thank you, we have now provided additional detail about the adaptations to the interview schedule, as outlined our response to reviewer 1, remark #9. Due to the length of the paper, we have provided the final tool in the appendix, instead of including a detailed description of the adaptations within the manuscript. The manuscript has now been edited to state:

(page 9) *Modifications included incorporating local terminology, improving in-depth probing, and asking specifically about the vaccination of ‘children with disability or special needs’. Additional prompts were introduced to evaluate immunization efforts targeting underprivileged or remote populations and outreach strategies such as door to door services, see S1 Appendix.*

27. Rapport-building and using body language to encourage participation are useful, but this part could be more concise.
    **Response:** Thank you, but we believe these details provide important information about the data generation process in accordance with reflexive thematic analysis reporting.
28. The data analysis section is thorough. Are there specific examples of how the researchers' cultural backgrounds and personal experiences influenced the coding and analysis process? Currently, the mention of reflexivity feels superficial.
    **Response:** Our discussion of reflexivity was intentionally concise, as the primary focus of this paper is immunization. However, we appreciate the opportunity to elaborate further while maintaining brevity. The discussion has been revised to include the following:

(page 24) *Throughout the research, our team engaged in continuous self-reflection, critically considering how our identities and experiences influenced our interpretation of the data. For instance, our team’s diverse cultural and disciplinary backgrounds enriched the analysis by providing culturally relevant insights and avoiding Western-centric misinterpretations. Team members with experience in Fiji and other low- and middle-income settings brought essential understanding of local health practices and community attitudes toward disability and vaccination. Disability researchers contributed a deep awareness of the barriers faced by people with disability, while vaccination experts provided valuable perspectives on public health strategies and vaccination dynamics. This reflexivity guided our decisions on setting boundaries for inclusion within individual themes, enabling us to identify and generate “themes-as-shared-meaning,” ^14 (pp342)^ leading to a rich and nuanced analysis.*

29. The findings highlight the challenges of vaccinating children with disabilities. While the quotes add depth, some are repetitive.
    **Response:** Thank you for highlighting potential repetition in our results. We have carefully reviewed the manuscript and are confident that the selected quotes offer rich descriptions that enhance the depth and transparency of our analysis, illustrating key themes and supporting our interpretations effectively and re-iterating the gaps and opportunities.
30. A key issue is healthcare workers' lack of confidence and knowledge in administering vaccines to this group, and more details on the necessary training would help.
    **Response:** Thank you, we have now clarified this in the opening paragraph of the Discussion:

(page 22) *We found that the vaccination of children with disability in Fiji was hindered by a lack of information and support for parents of children with disability, concerns about vaccine benefits and safety, healthcare workers’ lack of confidence and knowledge in administering vaccines to children with disability, and communication and trust issues with healthcare workers.*

We also agree that targeted training is essential for building healthcare workers' confidence and knowledge when administering vaccines to children with disabilities. We have expanded the conclusion to include the following:

(page 25) *Training should encompass strategies to address disability stigma, contraindications if any including vaccine safety, and clear protocols for communication and consent tailored to children with disability and their families. Additionally, training should equip healthcare workers with practical skills for adapting vaccination approaches to support children with varying mobility and communication needs.*

31. The informed consent issues, where vaccines are given without proper explanation, need stronger focus on improving communication.
    **Response:** Thank you, we have edited the second paragraph of the Discussion to address this point more specifically:

(page 23) *System and policy responses are needed,^15^ including funding prioritization for workforce growth, providing training about vaccine safety for children with disability, and improving communication practices to enhance informed consent processes. These steps will help create a more supportive environment for vaccination in this population.^12^*

32. The mention of policy changes to include children with disabilities is promising, but more specifics on how these will be implemented would add valuable context.
    **Response:** We agree with the reviewer however it is beyond the scope of this study and a little premature. The study team is currently looking for funding to undertake the design and implementation process for this. However, it is important to acknowledge that without recognition of the issue and inclusion through a policy, this is difficult to achieve.
33. The study highlights the psychological, social, cultural, and environmental barriers to vaccinating children with disabilities in Fiji. While the connection to global vaccine uptake literature is relevant, there could be more emphasis on actionable steps to improve healthcare workers’ communication and trust-building with families.
    **Response:** Thank you for highlighting this important aspect. We agree that actionable steps to enhance communication and build trust between healthcare workers and families is crucial for improving vaccine uptake among children with disabilities. We have now included specific recommendations for training that focus on strategies to address disability stigma, ensure vaccine safety for children with disabilities, and establish clear protocols for communication and consent tailored to these children and their families.
34. The role of disability service providers in promoting vaccination is well-supported, but the discussion around solutions like outreach programs and after-hours clinics could be more concise.
    **Response:** Thank you for this suggestion. We believe that providing detailed discussion on outreach programs and after-hours clinics is essential to illustrate the range of practical, context-specific solutions suited to this setting. We feel these examples emphasize the importance of accessible, community-based options that directly address the unique needs of families with children with disabilities in Fiji.
35. The study’s strengths, such as stakeholder involvement and reflexivity, are clear, though future directions could be more succinct.
    **Response:** We have revised this section of the Discussion to address comments made by other reviewers, including specifying the study limitations.
36. Overall, the discussion is comprehensive but would benefit from sharper focus on practical implications.
    **Response:** Thank you for this feedback. We have revised the conclusion to sharpen the focus on practical implications, including specific recommendations for healthcare professional training. These revisions emphasize actionable steps that can be taken to address identified barriers and improve vaccination accessibility for children with disabilities.

*(page 25) Training should encompass strategies to address disability stigma, contraindications, if any, including vaccine safety, and clear protocols for communication and consent tailored to children with disability and their families. Additionally, training should equip healthcare workers with practical skills for adapting vaccination approaches to support children with varying mobility and communication needs.*

37. There are some minor grammatical errors in the paper, and the use of the terms ‘disability’ and ‘disabilities’ is not consistent.

**Response:** Thank you for noting this inconsistency. We have searched the paper for the term ‘disabilities’ and replaced it with ‘disability’ in instances other than participant quotations. This resulted in the following edit only:

(page 3) *The findings from this study can inform strategic actions to overcome barriers to vaccination for children with disability, including strengthening existing vaccination programs, promoting greater equity in vaccination for children with disability in Fiji.*

# Reviewer #6

38. The authors should provide more details on the study area Suva–Nausori corridor in Fiji. Is this in the south of the country or the north or east? Is this a State in the country? This will help readers understand the nature of the context.

**Response:** Thank you, we have now re-worked this section to provide additional information about the study location:

(page 8) *This study was conducted in peri-urban and urban areas of the Suva–Nausori corridor. The Suva Nausori corridor is a 19km stretch between Suva (capital of Fiji) and Nausori on its north east. The study site was chosen in collaboration with Fiji's Ministry of Health and Medical Services and non-governmental partners to ensure representation from both rural and urban areas. Based on in-country unpublished data it is believed to be an area with high prevalence of functional impairments among children (6% in ages 2-4 and 9% in ages 5-17)^2^, as well high population density (approximately 164,000)^3^, and challenging access to routine healthcare services.*

39. How many participants were in each of the focus groups (5) since they have 22 participants in total? Global literature on conducting an FGD suggests a minimum of 5-6 participants and a maximum number of 12 participants?

**Response:** The focus groups had an average of 4 participants per session. We have identified the small focus group size as a limitation in the Discussion.

(page 25) *The focus groups were also small, which may restrict the diversity of viewpoints captured.*

40. How was the participants allocated to each FGD determined?
    **Response:** We have provided additional detail to explain how and why participants were allocated to their respective focus group:

(page 9) *On the advice of our stakeholder group, participants working at the same organization were allocated to separate focus groups, while people of similar hierarchical positions were grouped together to manage power dynamics. This approach allowed participants to engage more candidly without concerns about authority or judgement from higher-ranking colleagues and minimized the risk of self-censorship and inhibited discussion.*

41. Do the investigators think the number in each FGD is adequate?

**Response:** Each focus group had an average of four participants, which we acknowledge resulted in relatively small groups. This is termed ‘mini focus groups’^16^ and are used in instances where there is only a small potential pool of participants available, yet the research design requires a group format. The decision to proceed with small focus groups was also pragmatic, as scheduling challenges prevented some participants from attending on the day. Despite the smaller group size, we ensured that discussions were rich and interactive, allowing for in-depth exploration of participants' perspectives. Additionally, the smaller size allowed participants more time to share their insights, and we were able to capture a range of viewpoints that aligned with the study’s objectives.

42. The investigators should provide the codebook that was used in the content and thematic anaysis process as a supplementary file?

**Response:** We welcome the opportunity to explain our thorough qualitative analysis methods in more detail. We have explained in the manuscript that we used Braun and Clarke’s method for reflexive thematic analysis ^17, 18^. Reflexive thematic analysis does not involve the development of a codebook, and uses a collaborative, reflexive approach to analysis. Our analysis was rigorous and systematic.

43. Table 1 should be added to the supplementary files.

**Response:** We have not included Tables in the paper and are unsure what the reviewer refers to.

# Reviewer #7

This study conducted in the context of low and middle income country is one of its first kind to be studied and therefore has merit and add to the body of knowledge and have implications for policy planning.

The study is relevant and will add to the body of knowledge. The authors have rightly pointed out that vaccine coverage among the healthy population is a challenge, so therefore, vaccination uptake among children with disability/disabilities, particularly living in middle- and low-income countries face manifold challenges. The following are my comments on this paper:

44. Line 33 – 34 is not clear – “Understanding the attitudes and behaviors of health, disability, and community stakeholders towards vaccination uptake in these children is important. – Whose attitudes and behaviors? Compare Lines 96 – 98 and 104 and reword this sentence to reflect clearly that the perspectives of the stakeholders are being examined as stated in your topic of the study.

**Response:** This sentence has now been edited:

(page 3) *This study aimed to understand the social and behavioral drivers of vaccine uptake among children with disability in Fiji, from the perspectives of health, disability and community stakeholders.*

45. Line 83 – what was the reason for the recovery vaccination coverage in 2023? What did the literature state, need expansion on this statement.

**Response:** We believe this is beyond the scope of the manuscript and likely not relevant. For the reviewers noting:

The disruption to immunization programs during the pandemic has been reported globally. It was impacted by staff being diverted to COVID response, health facilities being closed as well fear amongst caregivers to visit health facilities (see for e.g., ^19^ .

46. Line 105 – there should be a period after the word ‘disability’ marking the end of the sentence. Otherwise, it is confusing to the reader.

**Response:** Thank you, this typo has now been corrected.

47. Line 106 – mention where and under which section the research design for the first part is explained. It is very vague.

**Response:** This section has been edited to improve clarity, and moved to the ‘design and theoretical framework’ section:

(page 7) *The study is part of a two part study looking at immunization in children with disability. Part 1 involved a cross-sectional survey examining vaccine uptake among 198 children with disability and their caregivers (published elsewhere, see ^1^). Part 2 involved a qualitative study utilizing focus groups with health, disability, and community stakeholders on the behavioral and social drivers of vaccination uptake among children with disability. In this paper, we present qualitative data from the focus groups.*

48. Line 108 – Under Design and Theoretical Framework, no comprehensive research design of the present study was stated or any theoretical framework included. This section needs more elaboration on the research design and theoretical framework. Only Vaccination Framework was mentioned but that too need some information for the readers who are not familiar with this framework.

**Response:** Thank you for noting this concern. We have now revised the ‘design and theoretical framework’ section to clarify our approaches better:

(page 7) *The study is part of a two part study looking at immunization in children with disability. Part 1 involved a cross-sectional survey examining vaccine uptake among 198 children with disability and their caregivers (published elsewhere, see ^1^). Part 2 involved a qualitative study utilizing focus groups with health, disability, and community stakeholders on the behavioral and social drivers of vaccination uptake among children with disability. In this paper, we present qualitative data from the focus groups.*

*A 12-member stakeholder group was actively engaged and involved throughout all stages of the project providing expertise to guide the study design, data collection, analysis, and dissemination of results.^4^ The group included local and international disability and health service providers (study partners), MHMS, academics, and community and faith leaders. The stakeholder group identified focus groups as the preferred method for data collection, as participants were anticipated to feel more comfortable sharing their opinions in a group setting, reducing the pressure of individual interviews or a survey, and enabling them to control their level of participation.^6^ Additionally, the group setting fosters interaction among participants, encouraging the exchange of ideas and yielding a deeper, richer understanding of sensitive and potentially taboo topics.^6^*

*We used the World Health Organization’s (WHO) Behavioral and Social Drivers (BeSD) of Vaccination Framework^7^ to provide a structured approach to understanding the complex factors influencing vaccination uptake among children with disabilities. This framework offered a comprehensive guide to examine not only individual beliefs and behaviors but also social, cultural, and systemic drivers that can impact vaccine acceptance and access. This framework is well-suited for informing strategies to improve equitable vaccine access and uptake in contexts with diverse social and healthcare challenges.^7^*

49. Line 199 – What is meant by the phrase “plenty coconut wireless around”? We know that with respect to the dissemination of information and misinformation during COVID-19 was rampant through all platforms of communication that cause fears of the “unknown’ among the population. So, what was the scenario prior to COVID-19 in terms of information relating to vaccination, what misunderstanding or miscommunication if any prevailed during that time? Did you find any literature that can be included here or in the introduction or discussion section.

**Response:** We acknowledge that misinformation has become rather topical in vaccination and is an emerging issue. However, the COVID19 context is significantly different to routine immunization (examined here); and is beyond the scope of this paper. It would require more nuanced and in-depth analysis. Prior to this – misinformation which is different to coconut wireless (which is more like Chinese whisper) has not been a major issue in Fiji as evidenced from their high vaccination coverage.

50. Line 218 – with reference to this statement, did you investigate what perspectives stakeholders held prior to COVID-19, did COVID experience escalate the fear of the unknown with regards to vaccination among children with disabilities?

**Response:** The impact of COVID-19 vaccination was not examined, as it falls outside the scope of this study. Additionally, the qualitative study design does not allow for empirical measurement of pre- and post-COVID-19 experiences. Interview data did not reveal significant differences or challenges related to the COVID-19 experience.

51. Line 435 – 436 – is there any literature to support this finding?

**Response:** The opening paragraph of the discussion provides an overview of our key findings, which we then expand on and contextualize with relevant literature throughout the following paragraphs.

52. Discussion Section – should address all major themes of the study and linkages between these themes should be evident – thinking and feeling, social process, motivation, practical – need to revisit what you stated in the introduction with regards to these themes.

**Response:** Thank you for this suggestion – however as is standard, we have discussed the major aspects of the paper. The purpose of the discussion is to highlight key results which we have sufficiently done so. None of the other reviewers have raised this as an issue.

**References**

1.	Jahan I, Vakaloloma U, Perera S, Tuibeqa I, Devi R, Volavola L, et al. Vaccination and its social and behavioural drivers of children with disability in Fiji (manuscript under preparation).

2.	Fiji Bureau of Statistics. Fiji Multiple Indicator Cluster Survey 2021, Survey Findings Report Suva, Fiji: Fiji Bureau of Statistics; 2022 [Available from: <https://mics-surveys-prod.s3.amazonaws.com/MICS6/East%20Asia%20and%20the%20Pacific/Fiji/2021/Survey%20findings/Fiji%202021%20MICS_English.pdf>.

3.	Fiji Bureau of Statistics. Population and housing Census 2017 2017 [Available from: <https://www.statsfiji.gov.fj/census-surveys/census-of-population-and-housing/>.

4.	Graham ID, Logan J, Harrison MB, Straus SE, Tetroe J, Caswell W, et al. Lost in knowledge translation: time for a map? J Contin Educ Health Prof. 2006;26(1):13-24.

5.	World Health Organization (WHO). TIP: tailoring immunization programmes 2019 [Available from: <https://www.who.int/europe/publications/i/item/9789289054492>.

6.	Stewart DW, Shamdasani PN, Rook DW. Group Dynamics and Focus Group Research. Focus Groups: Theory and Practice. 2nd ed: SAGE Publications, Ltd.; 2007.

7.	World Health Organization (WHO). Understanding the behavioural and social drivers of vaccine uptake WHO position paper – May 2022. Weekly Epidemiological Record. 2022;97(20):209–24.

8.	Cobos Munoz D, Monzon Llamas L, Bosch-Capblanch X. Exposing concerns about vaccination in low- and middle-income countries: a systematic review. Int J Public Health. 2015;60(7):767-80.

9.	Rendell N, Sheel M. Expert perspectives on priorities for supporting health security in the Pacific region through health systems strengthening. PLOS Glob Public Health. 2022;2(9):e0000529.

10.	O'Neill J, Newall F, Antolovich G, Lima S, Danchin M. Vaccination in people with disability: a review. Hum Vaccin Immunother. 2020;16(1):7-15.

11.	Lin C, Mullen J, Smith D, Kotarba M, Kaplan SJ, Tu P. Healthcare Providers' Vaccine Perceptions, Hesitancy, and Recommendation to Patients: A Systematic Review. Vaccines (Basel). 2021;9(7).

12.	Smith F, Perera S, Marella M. The Journey to Early Identification and Intervention for Children with Disabilities in Fiji. International Journal of Environmental Research and Public Health. 2023;20(18).

13.	Woolfenden S, Milner K, Tora K, Naulumatua K, Mataika R, Smith F, et al. Strengthening Health Systems to Support Children with Neurodevelopmental Disabilities in Fiji-A Commentary. Int J Environ Res Public Health. 2020;17(3).

14.	Braun V, Clarke V. One size fits all? What counts as quality practice in (reflexive) thematic analysis? Qualitative Research in Psychology. 2020;18(3):328-52.

15.	Brewer NT, Chapman GB, Rothman AJ, Leask J, Kempe A. Increasing Vaccination: Putting Psychological Science Into Action. Psychol Sci Public Interest. 2017;18(3):149-207.

16.	O.Nyumba T, Wilson K, Derrick CJ, Mukherjee N, Geneletti D. The use of focus group discussion methodology: Insights from two decades of application in conservation. Methods in Ecology and Evolution. 2018;9(1):20-32.

17.	Braun V, Clarke V. Using thematic analysis in psychology. Qualitative Research in Psychology. 2006;3(2):77-101.

18.	Braun V, Clarke V. Reflecting on reflexive thematic analysis. Qualitative Research in Sport, Exercise and Health. 2019;11(4):589-97.

19.	Randell M, Sheel M, Dynes M, Li M, Danchin M, Oktarinda, et al. Influence of the COVID-19 pandemic on caregiver beliefs and experiences of routine childhood immunisation in Indonesia. Vaccine. 2024;42(4):812-8.
